# Perspectives of Canadian fitness professionals on exercise and possible anorexia nervosa

**DOI:** 10.1186/s40337-015-0074-y

**Published:** 2015-11-17

**Authors:** Amy E. Wojtowicz, Angela S. Alberga, Colleen G. Parsons, Kristin M. von Ranson

**Affiliations:** Department of Psychology, University of Calgary, Calgary, Canada; Werklund School of Education, University of Calgary, Calgary, Canada; Health and Fitness Programs, Faculty of Kinesiology, University of Calgary, Calgary, Canada

**Keywords:** Anorexia nervosa, Exercise, Subclinical, Fitness, Survey, Ethics, Eating disorders

## Abstract

**Background:**

Many individuals with eating disorders, including anorexia nervosa (AN), engage in overexercise. Little is known about fitness professionals’ perceptions of their responsibilities when interacting with clients with possible AN. The purpose of the current study was to examine Alberta fitness professionals’ experiences with clients suspected of having AN, and their views on related ethical issues. Specifically, we aimed to examine (1) their experiences with fitness clients suspected of having AN; (2) their opinions about related ethical responsibilities of fitness professionals; and (3) their views on related training and ethical issues.

**Methods:**

We administered a 21-item online survey to 143 Canadian fitness professionals about their experiences and perspectives on encountering individuals with possible AN in exercise classes and at their exercise facilities.

**Results:**

Sixty-two percent of respondents had encountered a client they believed had AN. Three-quarters had never received any training on managing clients with AN and felt inadequately prepared for such situations. Although most felt ethically obliged to intervene with such a client, more than two-thirds reported no relevant guidelines in their professional training.

**Conclusions:**

Many fitness professionals are faced with clients with possible AN, have the desire to help, feel ethically obligated to take action, but do not know what course of action to take, if any. Work is needed to clarify ethical issues and related training needs for certification programs for fitness professionals regarding AN.

## Background

Anorexia nervosa (AN) is associated with impairment in psychological and physical health, including comorbid psychopathology, nutritional deficiencies, amenorrhea, bone thinning, cardiac problems and early mortality [1, 2]. In addition, excessive exercise is a frequent concomitant of AN [3]. Approximately 39–81 % of adult clients with AN engage in compulsive exercise, especially during acute phases of the disorder [4–6]. The terms most often used to describe problematic exercise are excessive exercise and compulsive exercise within the eating disorder literature. According to DSM-5 excessive exercise is “any exercise that significantly interferes with important activities, occurs at inappropriate times or in inappropriate settings, or continues despite injury or other medical complications” [3]. A review by Meyer et al. [7] emphasized the need to develop a definition of “compulsive exercise” tied to a clear theoretical model. These authors define compulsive exercise as a “condition characterized by an association with weight and shape concerns, and persistent continuation in order to: (a) mitigate the experience of extreme guilt and/or negative affect when unable to exercise; and (b) avoid the perceived negative consequences of stopping” [7], pp. 184). Additional terms used for problematic exercise were summarized by Manley et al. [8] include “anorexia athletica,” “exercise bulimia,” “exercise disorder,” “exercise dependence,” “exercise addiction,” “obligatory exercise,” and “overexercise.” In the absence of a consensus, in this paper we use the broad term “overexercise” to encompass these interrelated concepts.

Among individuals with AN, overexercise may lead to increased risk of overuse injuries and bone fractures [9], cardiac complications [10] and poor AN treatment outcomes [11]. Although moderate exercise may be safe and beneficial for clients with AN [12], individuals with AN who overexercise may experience adverse consequences. As a result, ethical questions arise regarding the appropriate role of a fitness professional in recognizing or managing AN in a client [13].

Giordano [13] argued that although there are strong ethical reasons to let participants with suspected AN to participate in exercise classes, there is no moral obligation to allow a person with suspected AN to take part in  exercise classes. In one of the few studies on this topic, Manley et al. [8] administered a survey including a case study of an individual with probable AN to 62 fitness instructors and 56 pediatricians in British Columbia, Canada. The authors evaluated participants’ capability of recognizing an individual with AN, participants’ understanding of the professional, ethical and legal issues and inquired what they would do professionally during such an encounter. The results showed that 60 % of fitness instructors identified that there were some ethical and liability issues in the case study and all fitness instructors requested guidelines to assist with decision making.

In a more recent study on this topic, Bratland-Sanda and Sundgot-Borgen [14] examined the knowledge and attitudes of 837 Norwegian group fitness instructors toward identification and management of disordered eating in clients. Only 47 % knew how to recognize and respond to disordered eating and 37 % knew the fitness centers’ guidelines for approaching disordered eating concerns. Only half felt confident on how to approach concerns about disordered eating among members (49 %) and among fitness center colleagues (47 %).

Although no published guidelines by any international sport or eating disorder organization exist on the management of fitness center clients with suspected eating disorders and/or overexercise, fitness organizations in Australia, the USA, the UK and Norway have published their own guidelines. In 2004, Australian guidelines for identifying and managing members with disordered eating were published by the Fitness Australia in collaboration with the Center for Eating and Dieting Disorders. These guidelines suggested that if a member is suspected of having disordered eating symptoms, a senior staff or management member must be notified who will then decide on what future actions to take [15]. A more recent resource was published in 2014 from The National Eating Disorders Collaboration (NEDC), an initiative of the Australian Government Department of Health [16].

Other publications have addressed related issues but have not provided a unified voice about the best means with which to address disordered eating symptoms, including overexercise, among fitness clients. In 2007, the American College of Sports Medicine published a US position paper to raise awareness of the female athlete triad [17]. In 2010, Giordano [18] also published UK guidelines similar to Marks & Harding’s Australian guidelines [15]. The first guidelines in Norway were created in 2010 by Bratland-Sanda in cooperation with the Norwegian Association of Fitness Centers and an eating disorder nongovernmental organization [19]. Although these Norwegian guidelines were in line with Marks and Harding’s [15], the Norwegian guidelines did not include information on how to communicate concern to the member with a suspected eating disorder.

More information is needed to help shape the development of appropriate international guidelines. Little is known about the perceptions of fitness professionals of their responsibilities when interacting with clients with possible AN. Furthermore, it is unclear how consistent fitness professionals’ views are across types of fitness professionals, settings and countries. The purpose of the current study was to examine Alberta fitness professionals’ experiences with clients suspected of having AN, and their views on ethical implications. Specifically, we aimed to examine: (1) their experiences with fitness clients suspected of having AN; (2) their opinions about the ethical responsibility of fitness professionals and; (3) their views on related training and ethical issues. Although overexercise has been identified as a significant factor in the cause, development and maintenance of various eating disorders [20], we elected to focus this study on AN because our clinical experience, and the experience of others [21], has suggested that exercisers who appear to be underweight may elicit greatest concern from others. When presented via vignettes, AN can be identified by some fitness professionals [8] and community members [22], and AN may be more readily identified than other eating disorders [23. In addition, given the high rate of mortality among those with AN [24], overexercise is especially concerning among people with AN.

## Method

### Participants

Individuals currently working as fitness professionals in Alberta were eligible to participate in this web-based survey. Fitness instructors, personal trainers, facility operators and administrators were recruited. There were no exclusion criteria. Five methods were used to recruit potential participants: (1) a link of our survey was posted on the Alberta Provincial Fitness Unit website, www.provincialfitnessunit.ca, a website for conveying news to fitness professionals; (2) 322 agencies listed in the fitness section on a public website, www.alberta.servpro.ca, were contacted by e-mail and asked to pass on the survey link to all fitness staff; (3) 108 agencies listed in the fitness section of the Calgary Yellow Pages were contacted by telephone and asked to pass on the survey link to all fitness staff; (4) one author (CGP) with professional links to Alberta fitness organizations e-mailed 28 professional contacts a link to the survey; (5) individuals who completed our survey were also invited to pass on the survey link to other eligible participants. In total, approximately 350 e-mail messages and 108 telephone calls were executed to solicit participation in the survey.

### Measures

A 21-item online survey containing four sections was developed for the study by AEW, KMvR and CGP. KMvR can be contacted for a copy of the survey. Section 1 included questions describing participants’ experiences with clients they perceived as possibly having AN (hereafter, “with possible AN”). The following brief description of AN symptoms was provided: “Anorexia nervosa is a psychological illness in which individuals starve themselves or use other techniques, such as vomiting, taking laxatives or excessive exercise, to induce weight loss. Individuals with anorexia nervosa have a false perception of their bodies as being fat and/or have an intense fear of becoming fat. The result is a severe and often dangerous loss of weight.” Participants were then asked if they had encountered one of more individuals with possible AN in their gym or exercise classes, and details and attitudes regarding any course of action taken. Respondents were instructed to answer the questions based on their most recent encounter with a client with possible AN. Section 2 included questions about participants’ opinions regarding fitness professionals’ role with clients with possible AN. Section 3 asked participants whether they had received any training on how to manage a client with possible AN and whether their governing code of ethics addressed the issue. Section 4 requested participants’ demographic information. Throughout the survey, participants selected responses from a list of supplied options and had the option of providing a qualitative answer if they wished. Participants were permitted to provide multiple responses to certain questions. To ensure participation was entirely voluntary and to minimize pressure to respond in socially desirable ways, participants were permitted to skip any question or to exit the survey at any point.

This study, which was conducted in 2006, was approved by the University of Calgary Conjoint Faculties Research Ethics Board (file #4713). Participation involved informed consent.

## Results

One hundred and fifty-four people clicked “accept” on the consent form page prior to seeing any of the online survey questions. Out of these, 10 people did not answer any of the survey questions and one person answered ‘yes’ to the first question “Have you ever encountered clients you felt might have had anorexia nervosa?” but did not answer any of the subsequent survey questions. Thus, a total of 143 surveys were analyzed. The average time to complete the survey was 14 min after outliers (e.g., those who took more than 2 h) were excluded. Sample sizes vary, as not all respondents answered every question. Of the 117 participants (mean age 31.7 ± 7.6 years) who supplied demographic information, 71.7 % were female, 89.1 % were Caucasian, and 76.3 % reported having received provincial or national professional certification from the Canadian Society for Exercise Physiology (CSEP), the Alberta Fitness Leadership Certification Association (AFLCA) and/or the American College of Sports Medicine (ACSM). Other forms of training including a bachelor’s degree in Kinesiology or Physical Education, Certified Fitness Professional by Canfitpro (Canadian Fitness Professionals) or specialized training in a specific type of exercise (e.g., Pilates, martial arts, yoga, ballet) were reported in 19.5 % of our participants. Total duration of training ranged from ≤1 year (29.6 %), 2–5 years (49.0 %) to more than 6 years (21.4 %). Total duration of employment in the fitness industry ranged from ≤1 year (32.5 %), 2–5 years (32.5 %) and more than 6 years (35.1 %). Occupations (*n* = 121) included personal trainer (44.6 %), fitness instructor (27.3 %), facility operator (16.5 %), administrator (11.6 %), manager/supervisor/fitness coordinator (8.3 %), kinesiologist/athletic or exercise therapist (5.0 %), and other (9.1 %). The “other” category included responses such as dance teacher, martial arts and yoga/Pilates. Categories of occupations do not total 100 % because 10.7 % of our participants indicated more than one fitness-related occupation. Out of the 20 participants who had a role as a ‘facility operator’, 70 % reported having certifications from organizations such as CSEP, AFLCA or ACSM while 15 % reported another type of certification or no certification (*n* = 3). Out of the 14 participants who had a role as an ‘administrator’, 78.6 % reported having certifications from CSEP, AFLCA or ACSM while 14.3 % reported another type of certification or did not respond to the certification question (7.1 %). Work setting (*n* = 120) included public (47.5 %), private (25.8 %), educational institution (20.0 %), corporate club (15.0 %), mobile (3.3 %) and other (2.5 %) (e.g., community, martial arts school, rehabilitation center).^1^

### Experience with fitness clients suspected of having AN

Survey results are summarized in Table 1. Critical findings are summarized here. Almost two-thirds of respondents reported having encountered a client with possible AN, and more than half took some form of action with this client. All who took action did so as they were concerned about the client’s safety and well-being. Another reason for taking action endorsed by more than three-quarters was that respondents believed it was their duty as fitness professionals to protect their clients. However, more than half of respondents who took action felt that their action was inadequate or ineffective.

**Table 1:** Fitness professionals’ responses to online survey questions regarding clients with possible anorexia nervosa

	*N*	%
*Section 1: Experience with Fitness Clients Suspected of Having Anorexia Nervosa*		
Have you ever encountered clients that you felt might have had anorexia nervosa?	143	
● Yes	89	62.2 %
If yes, how confident were you that the client had anorexia nervosa?		
● Extremely confident	33	39.8 %
● Moderately confident	36	43.4 %
● Mildly confident	14	16.9 %
Did you take action (with the client that you felt had anorexia nervosa)?	83	
■ Yes	49	59.0 %
■ No	34	41.0 %
If no, why did you *not* take action?		
■ Unsure the client had anorexia nervosa	21	61.8 %
■ Not your place to intervene	18	52.9 %
■ To respect the client’s autonomy	15	44.1 %
■ To avoid embarrassing the client	11	32.3 %
■ Didn’t know what to do	8	23.5 %
■ The client must assume responsibility for her or his own behavior	3	8.8 %
■ Clients were made aware of their personal responsibilities regarding safety prior to participating/attending	2	5.9 %
If yes, what course of action did you take?	49	
■ Spoke to the client about your concern	43	87.8 %
■ Talked to someone else about your concern	26	53.1 %
■ Gave the client information on seeking help	23	46.9 %
■ Spoke to the client’s family/friends about your concerns	16	32.7 %
■ Did not allow the client to continue to exercise in class/gym	8	16.3 %
Why did you take action?	49	
● Concerned about the client’s safety and well-being	49	100 %
● Fitness professionals have a duty to protect their clients	40	81.6 %
● Concerned about legal liability	8	16.3 %
● Worried what other clients would think	6	12.2 %
● Other	5	10.2 %
Do you believe your course of action was adequate/effective?	49	
■ Yes	21	42.9 %
*Section 2: Opinions about the Ethical Responsibility of Fitness Professionals*		
Do you believe that fitness professionals are ethically obligated to take action when they encounter a client who they feel might have anorexia nervosa?	133	
■ Yes	102	76.7 %
■ No	18	13.5 %
■ Only when client is <18 years	13	9.8 %
What course of action should fitness professionals take if the client is an adult? (*n =* 99 participants chose ≥ 1 response)		
● Speak to the client about your concerns	96	97.0 %
● Give the client information on how to seek help	76	76.8 %
● Talk to someone else (e.g., boss, colleague) about their concerns	40	40.4 %
● Speak with the client’s family/ friends about their concerns	18	18.2 %
● Not allow the client to participate in their exercise class/gym	11	11.1 %
● Other	15	15.2 %
*Section 3: Related Training and Ethical Issues*		
Did you receive any instructions on how to handle a client you felt had anorexia nervosa during your training or personal and professional development as a fitness professional?	128	
■ Yes	33	25.7 %
Do you feel that how to handle a client you were concerned had anorexia nervosa was adequately addressed in the applicable code of ethics for fitness professionals?	72	
■ Yes	21	29.2 %
Do you feel that you were adequately prepared to handle a client you were concerned had anorexia nervosa?	17	
■ Yes	40	34.2 %
What information or training might help you feel better prepared to deal with a client you suspect has anorexia nervosa in the future? (*n =* 120 participants chose ≥ 1 response)	120	
■ The most appropriate course of action	108	90.0 %
■ Treatment options and community resources available for clients with anorexia nervosa	106	88.3 %
■ Information on liability issues related to such situations	82	68.3 %
■ Information about the risks of exercise for clients with anorexia nervosa	77	64.2 %
■ How to identify clients with anorexia nervosa	73	60.8 %
■ Other	8	6.7 %

Of those who responded that their course of action was not adequate/effective, participants were asked “Why was your course of action not adequate/effective?” as an optional open-ended response. One participant noted: “Because in my experience, this disease is one of denial. There is extreme fear of having to change behavior (i.e., not be able to work out and of being MADE to gain weight). Just speaking to an individual is better than not doing anything but it’s very difficult to get a person with anorexia (who is over the age of 18) to seek treatment that results in changing their behavior.” Another participant noted, “We lacked the skills to reach these people. They needed more professional help that we could not supply.” A third participant suggested: “It may have been temporarily effective but the individuals have once again come to my attention I believe with the same issues/concerns. I do not have enough background to stop the problem entirely and can see a possible need in the industry in regards to follow up for us to handle recurring clients of this nature.”

When asked “Why did you not take action?” one participant stated, ”[A]s instructors, we are limited in our range of expertise and services.”

### Opinions about related ethical responsibilities of fitness professionals

Three-quarters of respondents believed that fitness professionals were ethically obligated to act when they encountered a client with possible AN, regardless of the client’s age. Most participants responded that, if they took action, they would do so by speaking to the client about their concerns, give the client information on how to seek help, or both. Fewer than half reported they would talk to someone else about their concerns.

### Related training and ethical issues

At least two-thirds of respondents reported they had never received training on how to manage a client with AN, did not feel this issue was adequately addressed in their professional code of ethics, and did not feel prepared to manage a client with AN. The most common responses regarding what information or training might be helpful were: obtaining information on the most appropriate course of action and on available treatment options and community resources; earning how to identify clients with AN; obtaining information on related liability issues, and; obtaining information about the risks of exercise for clients with AN.

## Discussion

The current study describes the experiences of 143 fitness professionals from across a Canadian province working with clients with possible AN. In their work, two-thirds of respondents reported having encountered one or more clients who they suspected of having AN. Most reported they had never received any instruction on how to identify or manage clients with an eating disorder, and felt ill-equipped to intervene. Similarly, most respondents felt that their professional code of ethics did not adequately address related issues, specifically that an unacknowledged mental health problem may lead to short- or long-term exercise-related health risks. Nonetheless, most respondents believed that they had an ethical obligation to intervene. Although over half took action when faced with a client with possible AN, most deemed their actions ineffective and reported feeling inadequately prepared to manage such situations in the future. These results are generally consistent with earlier research with group fitness instructors [14], as well as previous findings that although a majority of college athletic trainers believed it was their role to identify and help female athletes with eating disorders, half did not believe they could effectively offer support [25].

### Ethical and legal issues

Participation of people with possible AN in fitness centers requires further attention to the physical, ethical and legal risks associated with their engagement in exercise. Our results show that 76.7 % of our sample believed that fitness professionals are ethically obliged to take action when they encounter a client who may have AN. Similarly, Manley et al. [8] also showed that 60 % of fitness instructors recognized that there were ethical and legal issues related to a case scenario discussing an individual with AN.

Giordano [13] has proposed two overarching ethical considerations, namely respecting the client’s autonomy and maintaining beneficence with aims of protecting the client and maximizing long-term health. He proposed that excluding a client with an eating disorder from a fitness class or facility may have more deleterious effects because it may increase the client’s sense of lack of control, which is already quite pervasive in people with anorexia (i.e., obsessive-compulsive personality traits) [26] and may compromise their health over the long term. For example, although the majority of fitness professionals in this study wished to help a client with possible AN, almost 15 % felt that taking action with a client suspected of having AN would violate the client’s autonomy, highlighting the complexity of these issues. If our participants did intervene by speaking to one of their client’s family members or friends, we unfortunately did not collect information on whether they first requested the client’s consent. Indeed, the appropriateness of intervening with any individual client may vary according to the situation, including the eating disorder symptoms that are exhibited and reported, as well as their level of exercise. In addition, there is the risk of misidentifying someone as having possible AN.

Other ethical principles that should be considered are the protection of the client’s confidentiality, and limiting practice to areas of competence. It is imperative that fitness professionals realize their limitations and do not exceed their professional scope of practice [27]. Thus, the primary role of the fitness professional should be to express concern, encourage the client to become more educated about the topic, and provide appropriate referral information [28].

Fitness professionals need guidance in identifying and considering ethical and liability issues that arise with individuals who are overexercising. Issues of liability require legal consultation and may vary across geographic locations. Providing such information may help fitness professionals to be more confident in identifying clients at risk and referring them to appropriate treatment resources in their area. These findings highlight the importance of incorporating relevant principles into professional codes of ethics that fitness professionals should consider when faced with a client suspected of having AN.

### Educational training

Unfortunately, it is not uncommon for fitness professionals to find themselves in the confusing situation of being faced with clients with possible AN, having the desire to help, feeling ethically obligated to take action, yet not knowing what to do. Despite the belief that fitness professionals feel ethically obligated to intervene, our results showed that when fitness professionals did intervene, only 42.9 % believed that their course of action was adequate or effective. Bratland-Sanda’s study of Norwegian fitness instructors adds support to these results, showing that less than half of their respondents reported knowledge on how to recognize and respond to concerns about their clients with suspected eating disorders [14] as well as to provide education on recognition and management of eating disorders and exercise for fitness professionals in training and working in fitness centers.

There is a pressing need for training of fitness professionals about signs and symptoms of AN, other eating disorders and overexercise, as well as information on treatment options and community resources for their clients with possible AN. For example, it may be wise for fitness professionals to routinely assess in detail clients’ reasons for undertaking exercise, as eating disorder psychopathology is strongly associated with exercising that is done primarily or solely to change one’s weight or shape [7]. Furthermore, because exercise and eating psychopathology may reciprocally reinforce each other [7], fitness professionals need to be aware of trends in their clients’ activity level and weight.

Making information brochures available for fitness professionals and clients that include information on eating disorders and local treatment centers may be helpful. Several resources about medical risks associated with exercise for individuals with AN are readily available [29–31]. One may argue that individuals suffering from AN may have diminished capacity to deliberate rationally about exercise, depending on the degree of weight loss. It is also important to consider the potential negative impact that individuals with AN who are engaging in exercise may have on the exercise attitudes and behaviors of other clients utilizing the same fitness facility. For example, after observing an underweight individual working out excessively, at-risk clients may go on to set unrealistic and unhealthy goals for themselves. It may also be detrimental to clients with possible AN if their observations of other clients’ exercise behaviors result in them exercising more intensely or for longer durations than they would have otherwise.

### Practical applications

The present survey results clearly indicate that fitness professionals would like access to more information, guidance and training on treatment options and community resources available for clients with AN. A single course of action is unlikely to be appropriate in all situations, as a number of interpersonal and contextual factors can influence the outcome (e.g., the severity of a client’s weight loss, the intensity and frequency of the client’s exercise, the client’s awareness of their behavior, the extent and quality of the relationship between the fitness professional and the client). However, we propose the following suggestions for the roles of fitness professionals, the fitness facilities, training curricula and position statements for fitness professionals.

### Roles of fitness professionals

The first challenge for fitness professionals is to become familiar with symptoms of AN. Learning how to identify clients with AN is particularly important because an inability to accurately identify people with AN was one of the main reasons that fitness professionals reported not taking action in the past. Identifying clients with AN is not a simple task, as very thin people do not necessarily have AN [13], and people with AN may not appear skeletal. Misidentifying an individual could have serious consequences, as the client may feel discriminated against. One participant stated: “When I approached her husband about my concerns, he told me to mind my own business [;] he liked her that way. They were both long distance runners and he was on the thin side too. They stopped training at my club and I lost contact with her. I hope she took my advice and got help, but unfortunately her husband was a large part of the problem and she probably didn’t.” This example also illustrates a potential conflict of interest for fitness professionals because if they take action, they might risk losing a paying client. It is important to highlight this potential conflict of interest for fitness professionals to make a more informed decision. The financial welfare of the fitness professional or fitness facility should not take precedence over the welfare of the client.

The issue is further complicated by the fact that individuals with AN commonly deny their eating disorder symptoms [32]. In this study, for example, one participant noted, “The client didn’t really seem to care about my concerns, I was quickly brushed off and shortly after the client ended her sessions and we did not work together again. I am uncertain why she did not return.” Thus, without knowing an individual’s true reasons or motivation for being underweight, it is likely that fitness professionals will remain uncertain about whether any individual has AN or not, and so will need to decide whether and how to act on the basis of incomplete information. Similarly, there is ambiguity about what amount or kind of exercise, in what context, should raise a red flag to a fitness professional. A person whose exercise is not excessive may still be endangering their health if their weight is very low, for example.

It is also important that fitness professionals continue to act supportively and are caring even if a client suspected of having AN reacts defensively [28]. Identifying clients who are at risk, offering resources, maintaining confidentiality and providing support, are all potentially valuable steps in assisting clients suffering from AN [27], but the ultimate responsibility for change lies within the client.

Of course, fitness professionals are not immune to developing eating disorders themselves. In fact, previous research has shown that women with a history of an eating disorder are often attracted to professions in fitness [28]. More recently, Bratland-Sanda et al. showed that 29 % of their sample of Norwegian fitness instructors self-reported a history of an eating disorder [14]. Training and certification programs for fitness professionals are strongly encouraged to integrate education regarding moderate exercise, healthy eating patterns and warning signs of compulsive exercise and eating disorders [28]. Similarly, fitness professionals are encouraged to reflect on their own eating and exercise patterns and seek help as needed. To effectively help their clients, fitness professionals need to first demonstrate positive attitudes towards their own bodies [28].

### Roles of fitness facilities

In fitness facilities, fitness professionals can also strive to provide an environment that promotes healthy body image for all clients, for example by encouraging healthy physical activities, educating clients about the importance of adequate nutrition and health habits, and limiting the amount of time that clients may spend utilizing certain or specific equipment. More information on the prevention of eating disorders within fitness facilities can be found in Perkins [27]. We recommend that fitness professionals develop and implement eating disorder policies that are applicable to their particular work setting. A sample policy on eating disorders that was designed for collegiate athletic trainers can be found in Vaughan et al. [25]. Several principles that have been previously discussed in the context of non-negotiables in the treatment of AN (e.g., [33]) may also apply to developing eating-disorder policies in the context of a fitness facility. First, eating disorder policies should be implemented consistently across clients and across fitness staff. Second, as with other policies, it is beneficial to have all clients (regardless of risk) be made aware of policies regarding safe exercise when they first begin a relationship with the fitness professional or fitness organization, to minimize the element of surprise if the policy is implemented. Fitness professionals are encouraged to invite clients to share their reactions to the eating disorder policy and answer any questions prior to participating in physical activity. Third, fitness professionals need to be informed about the rationale behind the eating disorder policy, and should be able to explain the rationale to clients without discomfort. Fourth, facility operators should periodically take the time to reflect upon whether their eating disorder policy is working and is being implemented fairly. If not, appropriate changes should be made.

### Training curricula and guidelines

The development of position statements and guidelines for Canada and other jurisdictions regarding identification and management of members with eating disorders and/or problems with overexercise are warranted. Our results highlight the need to include education, training, skills and competencies regarding excessive exercise and eating disorders in the curricula of Canadian fitness professionals. Bratland-Sanda et al. [14] also showed that exercise-specific education and instructor experience were explanatory variables for the fitness instructors’ knowledge of how to manage clients with an eating disorder. Specifically, fitness professionals should be able to recognize signs and symptoms of an eating disorder, overexercise and know what steps to take if any of these conditions are suspected.

### Strengths and limitations

Although the current study has important implications for training and certification programs for fitness professionals, we acknowledge certain study limitations. First, as one of the first studies on this topic, it is primarily descriptive in nature. Second, we do not know how representative the study’s sample was of fitness professionals in Alberta in 2006, or whether trends may have changed since then. We speculate that no substantive differences would be found if the study were to be repeated today. No Canadian guidelines have been created related to identifying or recognizing excessive exercise and disordered eating symptoms among individuals exercising in fitness facilities, so in this regard the environment is similar now as it was in 2006. Although a large number of e-mails and telephone calls were executed to launch the survey, the response rate was relatively low (approximately 31.2 % of 458 contacts made). Third, missing data from skipped questions and incomplete surveys limit the conclusions that can be drawn from the study. Nevertheless, a significant number of Alberta fitness professionals participated, representing a range of occupations and work environments. Fourth, social desirability may have played a role in participants’ responses when they were answering controversial questions. That is, what fitness professionals said they would do may differ from what they would actually do in a particular situation. Fifth, we cannot confirm that the clients that respondents suspected of having AN did in fact have this disorder. We relied upon participants’ reports so were unable to determine the accuracy of the participants at identifying those with possible AN. However, this problem may be similar to real life: fitness professionals may often find themselves unable to confirm if someone they suspect may have AN really has the disorder.

### Future research directions

More research is needed to explore this complex issue. Information about fitness professionals’ personal history of an eating disorder or other mental illness was not acquired. It may be particularly valuable to invite individuals who have recovered from AN or other eating disorders to discuss the recommendations that they would give to fitness professionals on this sensitive topic. It would also be valuable to survey the opinions of mental health professionals (i.e., psychologists, psychiatrists, social workers, etc.) who treat clients with AN to understand their perspectives on the role that fitness professionals may play in identifying and treating eating disorders. Future research should further explore issues related to fitness professionals and eating disorders, including in other jurisdictions and investigate the boundaries of moderate exercise and overexercise, and indicators of when a client with an eating disorder should not exercise.

## Conclusion

The findings of the study showed that most of the Canadian fitness professionals surveyed had encountered a client with possible AN but felt unprepared to manage the situation. The majority of our participants felt ethically obliged to help in some capacity because of their concern for their client’s safety and wellbeing. Our results highlight the importance of providing more information, resources, guidance and training to Canadian fitness professionals about how to manage clients with possible AN.

## Footnotes

1 In post hoc analyses we identified no gender, occupational or work setting differences in whether a fitness professional reported having encountered a client with possible AN, their confidence level in whether the person had AN, the number of reasons they thought the client had AN, the gender of the client with possible AN, whether the client with possible AN was under 18, or whether they took any action about a client with possible AN (*p* > 0.05).
